# Immunomodulatory Effects of IL-2 and IL-15; Implications for Cancer Immunotherapy

**DOI:** 10.3390/cancers12123586

**Published:** 2020-11-30

**Authors:** Ying Yang, Andreas Lundqvist

**Affiliations:** 1 Department of Respiratory, The Fourth Affiliated Hospital, Zhejiang University School of Medicine, Yiwu 310009, China; 11618300@zju.edu.cn; 2 Department of Oncology-Pathology, Karolinska Institutet, S-17164 Stockholm, Sweden

**Keywords:** cytokine, interleukin-2, interleukin-15, immunotherapy

## Abstract

**Simple Summary:**

Emerging knowledge of the two type I cytokine family members IL-2 and IL-15 has led to critical therapeutic implications for cancer treatment. Here we discuss the distinct roles of IL-2 and IL-15 in activating various functions of T and NK cells with a particular focus on the signals that contribute to the resistance of immune suppressive factors within the tumor microenvironment. We furthermore highlight efforts to modify these cytokines to amplify their antitumor efficacy while minimizing toxicity. Finally, we summarize the clinical applications of IL-2 and IL-15 in metastatic cancer.

**Abstract:**

The type I cytokine family members interleukin-2 (IL-2) and IL-15 play important roles in the homeostasis of innate and adaptive immunity. Although IL-2 and IL-15 receptor complexes activate similar signal transduction cascades, triggering of these receptors results in different functional activities in lymphocytes. While IL-2 expands regulatory T cells and CD4+ helper T cells, IL-15 supports the development of central memory T cells and NK cells. Recent data have provided evidence that IL-2 and IL-15 differ in their ability to activate T and NK cells to resist various forms of immune suppression. The diverse roles of these two cytokines have on immune cells lead to critical therapeutic implications for cancer treatment. In this review, we discuss the distinct roles of IL-2 and IL-15 in activating various functions in T and NK cells with a particular focus on the signals that participate in the resistance of tumor-derived immune suppressive factors. Furthermore, we summarize current clinical applications of IL-2 and IL-15 in metastatic malignancies, either as monotherapy or in combination with other agents, and highlight the future trends for research on these cytokine-based immunotherapies.

## 1. Introduction

The immune system is a complex network dedicated to protecting an organism against harmful substances, including the eradication of invading pathogens or malignant cells, maintenance of specific memory lymphocytes and elimination of autoreactive immune cells to yield self-tolerances. Homeostasis of immune systems relies on two main components—the innate and adaptive immune responses, which are regulated by a series of cytokines that are released in response to certain stimulus. One of the most extensively studied cytokines is the common cytokine receptor common gamma chain (γc) family of cytokines, including interleukin-2 (IL-2), IL-4, IL-7, IL-9, IL-15 and IL-21, which is named based on the usage of γc subunit for their receptors. This set of cytokines display broad pleiotropic actions to regulate both the innate and adaptive immune system, collectively contributing to the development of various immune cell populations, modulating cell differentiation, and either promoting the survival or inducing the apoptosis depending on the cellular context [1]. IL-2 is the first member of this family to be discovered with a vital role in T cell development and expansion; IL-15 was later identified to share a number of biological activities with IL-2, which include stimulation of the proliferation and activation of T cells and NK cells, induction of B cell immunoglobulin synthesis and supporting cytolytic effector cell differentiation [2]. These redundancies could be explained by the common receptor subunits contained by receptors for IL-2 and IL-15, the shared β chain and γ chain, which trigger similar intracellular signaling pathways following binding with IL-2 or IL-15 [2]. Despite these similarities, IL-2 and IL-15 also display distinct functions in vivo, especially in adaptive immune responses. For example, IL-2 is required for the development and persistence of regulatory T (Treg) cells, and it is crucially involved in activation-induced cell death (AICD). By contrast, IL-15 is the major force for supporting natural killer (NK) cells and memory CD8+ T cell persistence, while its precise role with regard to Treg cells is still controversial [3]. It does not mediate AICD, but instead inhabits AICD induced by IL-2 [4]. In addition, recent studies have revealed the different ability of IL-2 and IL-15 to facilitate the activation and persistence of T and NK cells against various immune suppressive factors [5,6,7]. Their private receptor component, IL-2Rα or IL-15Rα, might contribute to these distinctive functions of IL-2 and IL-15, but further investigations are still required for better understanding of mechanism behind their differences.

Immunotherapy has improved the treatment outcomes in patients with cancer, and continuous efforts are devoted to exploiting the therapeutic potential of IL-2 and IL-15 based on their ability to expand and activate cytolytic lymphocytes in vivo. The utility of IL-2 as an antitumor agent was approved by the FDA in patients with advanced melanoma and renal cell carcinoma (RCC) decades ago [8]. Despite the encouraging clinical response rate, the accompanying severe side effects and toxicity of IL-2 therapy remains a major limitation. IL-15 has emerged as an alternative to IL-2 in cancer treatment, for its potent effects on cytolytic NK and T cells without inducing suppressive Treg cells. More recently, increasing insights on the biology of IL-2 and IL-15 have allowed remarkable translation advances in modulation of the pharmacokinetics of these cytokines to bypass limitations and boost efficacy. Meanwhile, there is a growing focus on using cytokines in combination strategies for synergistic immune enhancement. Here, we summarize the current knowledge about the biology and function of IL-2, IL-15 and their receptor systems, with further discussion on clinical applications of these two cytokines for cancer treatment.

## 2. Overview of IL-2-IL-2R and IL-15-IL-15R System

### 2.1. The Biologic Profiles of IL-2 and IL-15

IL-2 and IL-15 are type I four α-helical bundle cytokines, referred to as the common γ receptor family of cytokines. This set of cytokines share the same receptor subunit γc and exhibit pleiotropic effects to modulate both innate and adaptive immune responses.

IL-2 was the first cytokine of this family to be identified; it was initially discovered from the supernatants of activated human T cells culture, which was a soluble factor that mediated T cell proliferation [9]. IL-2 is also the first cytokine approved by the FDA to be used in cancer treatment. While predominately secreted by CD4+ and CD8+ T cells following stimulation with antigen [10,11], a lesser amount of IL-2 is also produced by activated dendritic cells (DCs) [12], mast cells [13] and NKT cells [14]. Taking the advantage of Immgen data, Crellin et al. showed that ILC2 and ILC3 also produce *il2* mRNA [15]. IL-2 conditionally deleted mice were recently generated, which can lead to better understanding of cellular sources of IL-2 within different tissues under certain conditions [16]. Transcription factors activated by the signals from T cell receptor (TCR) and other costimulations, including NF-κB [17], NFAT family protein [18], OCT-1 [19], FOS and JUN [20], directly triggered the activation of the *Il2* gene [10,19]. As an autocrine/paracrine cytokine, the expression of IL-2 in T cells highly depends on the transcriptional regulation and mRNA stabilization, as well as the cellular activation state [21].

*IL-15* first reported by two independent groups as a T cell proliferation factor, IL-15 exhibited its capability to mimic the IL-2-stimulated growth of T cells [22,23]. Through the signals emanated from their shared receptor subunit, IL-2/15 Rβ and γc, IL-15 also shares certain similar functions to IL-2, which include stimulation of the activated T cell proliferation, generation of cytotoxic effector T cells and the activation and persistence of NK cells [24]. They also facilitate the induction of immunoglobulin synthesis by B cells [25] and the regulation of lymphoid homeostasis [26]. However, unlike IL-2, IL-15 mRNA expression was detected in various tissues, both in hematopoietic and non-hematopoietic cells such as keratinocytes, nerve cells, stromal cells and fibroblasts [24,27]. Different from the widespread IL-15 mRNA expression, mature IL-15 protein production is mainly limited to DCs and monocytes/macrophages [1,2]. There are two isoforms of IL-15 mRNA with different signal peptides lengths, although those two isoforms yield same mature IL-15, they have distinct effect on the intracellular trafficking and secretion of IL-15 [28,29,30]. This indicates that IL-15 protein production is primarily controlled by the post-transcriptional stage, mainly the translation and intracellular trafficking process [30].

### 2.2. Receptors for IL-2 and IL-15

**Figure 1:**
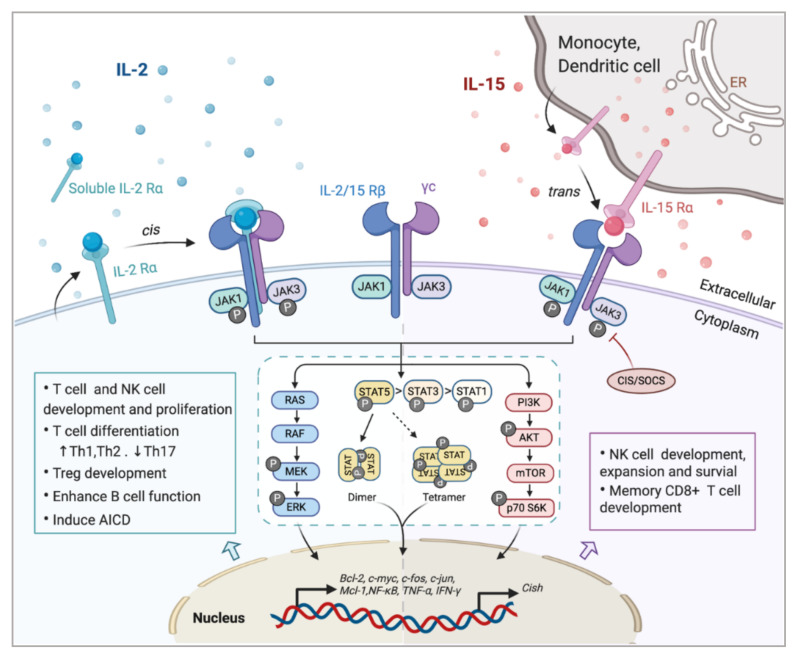
Interaction of interleukin-2 (IL-2) and IL-15 with their receptors and downstream signaling pathways. Receptors for IL-2 and IL-15 share two mutual submits, the common cytokine receptor γ-chain (γc) and the β-chain IL-2/15Rβ. Secreted IL-2 binds to its unique receptor submit IL-2Rα while membrane-associated IL-15 is trans-presented by monocytes or dendritic cells to NK cells or CD8+ T cells through binding with IL-15Rα, and then forms the high-affinity heterotrimeric receptor complex allowing the activation of downstream signaling. Both IL-2 and IL-15 mainly trigger the phosphorylation of the Janus kinase (JAK)/signal transducer and activator of transcription (STAT) pathway, which could be limited by CIS/SOCS as a negative feedback. The phosphorylated STAT dimers or tetramers then translocate into the nucleus to regulate the transcription of target genes. Other pathways including RAS/Raf/MAPK and phosphoinositol 3-kinase (PI3K)/AKT are also activated by the ligation of these cytokines, contributing to the complex physical impacts of IL-2 and IL-15 on various immune cells.

Investigating the components of receptor complexes and their downstream signals is essential to understand the biological effects of cytokines on the immune system. The receptors for IL-2 and IL-15 are both heterotrimeric, except for the common usage of cytokine receptor subunit, γc (also known as IL-2Rγ or CD132); they also share the beta subunit, referred to here as IL-2/15Rβ (also known as CD122) [31]. The third and unique receptor subunit for IL-2 and IL-15 is IL-2Rα and IL-15Rα, respectively (Figure 1). Most IL-2/15Rβ and γc are restrictively expressed by lymphohematopoietic cells, including T cells, NK cells, monocytes and neutrophils [10,32]. However, human fibroblasts were also reported to express functional IL-2/15Rβ and IL-2Rα, but the implications of this expression still remain unclear [33]. Ets1 family proteins, as well as Egr and Sp1, bind to IL-2/15Rβ promoter and trigger the expression in T cell and NK cells [34,35]. IL-2/15Rβ expression can be further regulated by IL-2, IL-4, PMA and TCR stimulation in both transcriptional and post-transcriptional phase [19]. The expression of γc has been shown to be boosted by IFNγ and IL-2 in monocytes whilst inhibited by TGF-β1 [36]. A post-transcriptional mechanism that mediates the soluble extracellular γc domain production and secretion in activated T cells has also been reported [37]. Although ubiquitination and cytokine-induced internalization evidently affect γc expression, little information is available concerning the cellular mechanisms controlling γc expression [38].

The surface receptor complex for IL-2 signal consists of various combinations of the three IL-2R subunits. By stimulation via TCR or cytokines including IL-2, IL-7, IL-12, IL-15, the cytokine-specific IL-2Rα (CD25) is transcriptionally induced on T cells and NK cells, while is minimally detected on resting cells [19]. IL-2Rα is also found to be expressed by myeloid dendritic cells [39]. The isolated IL-2Rα binds IL-2 with low affinity (K_d_~10^−8^ M) and serves only as a cytoplasmic anchor without transducing intracellular signals [10,40]. The combination of IL-2/15Rβ with γc forms intermediate-affinity heterodimeric receptor (K_d_~10^−9^ M), while all three subsites together bind IL-2 with high affinity (K_d_~10^−11^ M) and transduce intracellular signals [40,41]. In addition to the cell surface expression, IL-2Rα was also detected in certain diseases as in a soluble form (sIL-2R), including inflammatory disorders, transplantation rejection and most malignancy diseases [10,42,43]. The elevated level of sIL-2R ins serum is associated with disease progression and prognosis [10,43,44].

As the unique component of the IL-15 receptor complex, IL-15Rα is predominantly expressed on monocytes and dendritic cells, independent of IL-2Rβ and γc [2]. In contrast to IL-2Rα, IL-15Rα binds with IL-15 with high affinity by itself (K_d_~10^−11^ M) and potentially mediates certain intracellular signals [45,46]. Unlike the other γc family cytokines which function as soluble ligands binding to and acting on the receptor expressing cell in *cis*, IL-15 primarily signals in *trans* as a cell-associated cytokine bounds to IL-15Rα expressing cells. The IL-15/IL-15Rα complex is then presented to IL-2/15Rβ and γc on bystander activated T cells or NK cells to form high-affinity immunological synapse, with signals induced similar to IL-2R [47,48]. Although IL-2 can be trans-presented by some IL-2Rα expressing DCs [49], it mainly signals in *cis*.

### 2.3. Common Downstream Pathways of IL-2 and IL-15 Receptors

In light of the shared receptor subunits (IL-2/15 Rβγ), IL-2 and IL-15 trigger several similar downstream signaling pathways including activation of common Janus kinase (JAKs)/signal transducer and activator of transcription (STATs), with JAK1 interacting with IL-2/15 Rβ and JAK3 with γc (Figure 1). STAT proteins, primarily STAT5A and STAT5B, are recruited to dock on IL-2/15 Rβγ, where they get phosphorylated, form dimers and translocate to the nucleus to bind with target genes [50,51]. Moreover, the N-terminal region of STAT5 can mediate oligomerization of dimers to allow the formation of tetramers and binding to tandem motifs, which is critical for IL-2-induced early cytokine responses and IL-15-induced NK cell maturation and survival [51,52]. Beyond the activation of JAK/STAT signaling pathway, IL-2/15R complex also mediate the stimulation of phosphoinositol 3-kinase (PI3K)/AKT pathway to promote cell survival and proliferation via subsequent mTOR activation [53]. Additionally, signaling through IL-2/IL-15R complex induces the expression of antiapoptotic protein Bcl-2 and activation of the RAS-Raf-MAPK pathway which regulate the transcription factor complexes containing FOS/JUN [54]. Among all those signaling pathways, phosphor-proteomic analysis of activated T cells suggested that 90% of IL-2-induced signaling is JAK kinase dependent [55]. It has also been shown that IL-15Rα could interact with TRAF2 to act on the transcription factor NF-κB [56], but further studies are still required to understand the intracellular trafficking of IL-15Rα. Apart from positive signals, IL-2 and IL-15 also induce the expression of negative regulators to prevent excessive responses. For example, the suppressors of cytokine signaling (SOCS) proteins (CIS, SOCS1) inhibit the JAK enzymatic activity via directly binding to JAK1, JAK2 and TYK2, to form a negative feedback loop limiting the cytokine signaling, thus SOCS protein has been considered as a novel checkpoint for NK cell immunotherapy [57,58].

## 3. Immunomodulatory Effects of IL-2 and IL-15

### 3.1. Physiological Functions of IL-2 and IL-15

The common γ receptor family of cytokines collectively act to modulate development, proliferation, differentiation and survival of immune cells. Initially discovered as lymphocyte growth factor affecting the adaptive immune system, it has become clear that these cytokines are also involved in the innate immune response. Both IL-2 and IL-15 are critical regulators for innate lymphoid cells [59,60]. For example, IL-2 promotes elimination of pathogens by neutrophils, and IL-15 mediates the differentiation CD8αα+ intraepithelial lymphocytes via T-bet [61]. IL-2 and IL-15 mediate several similar functions on immune modulation as a consequence of sharing common receptor components and the JAK/STAT signaling pathway. These functions include the ability to promote proliferation and activation of CD4+ and CD8+ T cells, induce the differentiation of T helper cells and augment immunoglobin synthesis by activated B cells. Moreover, these two cytokines also play a crucial role in the generation and persistence of NK cells, potentiating the cytolytic activity of NK cells and CD8+T cells.

Despite these similarities, there are distinct differences between IL-2 and IL-15 with the actions on adaptive immune response (Figure 1). Apart from acting as a T cell growth factor, IL-2 also has a role in eliminating self-relative T cell via AICD [62], which is closely associated with the pathologic process of autoimmune diseases. In several systems, IL-15 has proven to be an antiapoptotic factor with potential to inhibit IL-2-induced AICD in vivo [4]. IL-2 usually favors the rapid proliferation of short-lived effector cells, while L-15 has its own unique effects on supporting the maintenance of long-lived memory phenotype CD8^+^ T cells and NK cells [63,64]. Although there have been a few reports showing that IL-15 is also involved in the development of Foxp3+ Treg cells [3,16,65,66], IL-2 is still recognized as the dominant driver for Treg cell development, homeostasis and fitness maintenance [67,68]. The capacity of IL-2 to activate both cytotoxic effector cells and Treg cells makes it a double-edged sword when utilized as an immunotherapeutic agent. To avoid this problem, different doses of IL-2 could be used based on the distinct IL-2R expression pattern on those two cell types. Compared with effector T cells, Treg cells express relatively higher levels of CD25, leading to a more frequent formation of high-affinity receptors for IL-2. As a result, while a high dose of IL-2 is required to preferentially expand effector T cells, Treg cells are able to respond rapidly to IL-2 at low concentrations (at single doses from 0.33 to 4.5 million IU) [69,70]. Additionally, the presence of IL-15 stimulates the production and recruitment of intestine intraepithelial γδ T cells; these results have not been observed with IL-2 [71,72]. Ex vivo cultured γδ T cells in the presence of IL-15 displayed prolonged survival and improved effector functions, as compared with IL-2. The production of αβ T cells from progenitor cells can be shifted to NK cells to a higher degree when encountering a high level of IL-15 [73]. Phenotypic differences such as cell size caused by IL-2 or IL-15 stimulation have less effect on signal transduction but more on the intensity and duration of the receptor signal [74].

Different transgenic mouse models have been used to confirm ex vivo functional observations of IL-2 and IL-15. IL-2 Rα deficient mice were more prone to develop autoimmune diseases, such as inflammatory bowel disease [75,76,77]. Massive enlargement of peripheral lymphoid organs associated with no selective T cell and B cell expansion was observed in IL-2 and IL-2 Rα deficient mice, due to the impairment of AICD and inhibited Treg cell development [78,79]. However, in mice with IL-15 or IL-15 Rα deficiency, no increased incidence of lymphoid enlargement or autoimmune disease was observed. Instead, they had remarkable reductions in NK cell, NKT cell and CD8+ memory T cell numbers in both periphery and thymus [80,81]. In the absence of NK cells and CD8+ memory T cells, IL-15-/- mice were more susceptible to various pathogens due to the compromised defense response. These selective lymphoid deficiencies could be reversed upon exogenous IL-15 provision, which further supports the critical biological role of this cytokine [81]. Trans-presentation of IL-15 mediated by IL-15Rα on antigen-presenting cells, such as DCs, is required for the generation and survival of NK cells, as well as for the longevity and avidity of antigen-specific CD8+T cells [47,48,64].

### 3.2. Immunomodulation of T and NK Cells in the Tumor Microenvironment

The development of tumors is often accompanied with an immunosuppressive microenvironment, hampering effector functions of cytotoxic lymphocytes, mostly CD8+ T cells and NK cells, to escape from immunosurveillance and promote progression. Overcoming the immunosuppression with sustained cytolytic activity of T and NK cells is required for efficient eradication of tumor cells. Recent studies have demonstrated different capacities of IL-2 and IL-15 in altering the susceptibility of T and NK cells to diverse immune suppressions. Discerning the roles of IL-2 and IL-15 in the regulation of antitumor immune responses is critical for the development of immunotherapeutic approaches against cancer.

*CD8+T cells*: Although IL-2 and IL-15 share many identical functions in the regulation of T cell as mentioned above, the distinct actions of these two cytokines on CD8+ T cells have been increasingly revealed. For instance, IL-15 is more efficient than IL-2 in cooperating with IL-21 to boost the expansion and effector function of splenic CD8+T cells, while for antigen-stimulated CD8+ T cells, IL-2 shows more potency in promoting protein synthesis than IL-15 [10,82]. The pivotal roles of IL-2 and IL-15 in activating CD8+T cells lead to the wide usage of these two cytokines in cancer immunotherapy. The in vivo persistence and activation of adoptively transferred T cells is usually maintained by IL-2 infusion, but with IL-15 as exogenous supplement or as transgene expressed, preclinical mouse studies demonstrated an enhanced antitumor capacity of CD8+T cells compared with IL-2 [83]. Recent studies have shown that IL-2 and IL-15 both triggered CD8+ T cell exhaustion by similarly inducing the expression of inhibitory receptor in vivo, particularly 2B4 and TIM-3, and selective abrogation of their common IL-2Rβchain could retain the inhibitory receptor induction [84]. In breast cancer, IL-15 provoked higher proliferation and IFNγ production of tumor-infiltrating CD8+ T cells than IL-2, and these strong but short-lived response could be diminished by the subsequently upregulated TIM-3 [85].

*NK cells:* The NK cell is a fundamental member of innate lymphocytes that mediates rapid and vigorous immunity against tumor cells. In adoptive NK cell therapy, both IL-2 and IL-15 can be used for the expansion and activation of NK cells in vitro before transfer. Although IL-2 is the most widely used in the clinic, IL-15 stimulation is reported to enable NK cells showing superior cytolytic performances and induce a memory-like NK cell population. In addition, IL-15 priming significantly ameliorated the antitumor response of CD56 bright NK cell subset [86]. Several studies have revealed the close association between IL-15 and the metabolic checkpoint kinase mTOR. Compared to IL-2, IL-15 augments stronger mTOR signaling, which is essential for the development and effector function of NK cells [87,88]. Meanwhile, continuous IL-15 exposure to NK cells could result in arrested cell cycle, diminished viability, reduced tumor cytolytic activity and metabolic deficiency, and this exhaustion status could be reversed by mTOR inhibitor [89]. TIM-3 could be induced on NK cells following the stimulation of IL-15 as well, which marks the maturation and cytotoxicity suppression status of NK cells [90].

In the context of supporting immune cell persistence in the immunosuppressive tumor microenvironment (TME), IL-2 and IL-15 have different potency in terms of regulating signaling pathway and protein synthesis. When encountered with abundant reactive oxidative species (ROS) in solid tumors, studies have shown that IL-15 stimulation upregulated the thioredoxin system in NK cells and T cells to confer increased tolerance towards oxidative stress [5,7]. As a complement, we found that IL-15 enhanced mTOR activity leading to higher levels of surface thiols on NK cells to neutralize extracellular ROS, compared with IL-2 [6]. Meanwhile, it has been reported that TGFβ could inhibit the activation and function of NK cells through curbing the IL-15-induced mTOR pathway [91]. These data indicate that IL-15 could be used as a promising antitumor agent to overcome immunosuppression in the TME.

### 3.3. Potential Mechanisms for the Distinction of IL-2 and IL-15

As the distinctive roles of IL-2 and IL-15 have been identified, it is vital to understand the potential mechanism underlying these contrasting functions. One factor is that these two cytokines are synthesized and secreted by different cells and tissues, which are regulated by distinct modes. Another reason could be the distinct ways these two cytokines interact with their receptors. As mentioned above, receptors for IL-2 and IL-15 comprise two subunits in common and mediate similar pathways including JAK/STAT, but they both have their private components, IL-2Rα and IL-15Rα, respectively. This means that the diverse physiological distribution of these two α-chains could also contribute to the biological differences of IL-2 and IL-15 in vivo. Moreover, a recent study has suggested that exposure to IL-15 causes the reduction in expression of IL-15Rα [48]. Apart from the similar signaling pathways, there are still several distinct downstream pathways through the receptor that have been detected. For example, T cell proliferation induced by IL-15 largely depends on FKBP12-mediated activation of p70S6 kinase, but FKBP12 is not indispensable for IL-2-induced proliferation [92]. Instead, the response of T cells to IL-2 requires another protein FKBP12.6, which is not involved in the response to IL-15 [2,92]. Additionally, as mentioned above, IL-15 triggers elevated mTOR signaling in NK cells compared with IL-2. To date, the molecular basis underlying the differences between IL-2 and IL-15 intracellular signaling has been poorly described; these preliminary findings require further investigations for optimizing clinical implications of IL-2 and IL-15.

## 4. Implication for Cancer Immunotherapy

Based on the discovery of the vigorous antitumor capacities of IL-2 and IL-15, multiple clinical strategies are currently being developed involving the use of cytokines for the treatment of several malignancies, as monotherapy or in combination with additional antitumor agents, with a few approaches that have already received approval from the FDA. Given the inadequacy of usual recombinant cytokines, considerable efforts are also being devoted to the improvement of novel cytokine agonists or immunocytokines. Here, we discuss recent clinical and preclinical advances of IL-2- or IL-15-based immunotherapy in cancer patients.

### 4.1. Clinical Applications of IL-2 and IL-15 as Monotherapy

The antitumor effect of IL-2 resulting from its capacity to expand and activate effector lymphocytes in vivo has been translated into the first available cancer immunotherapy. As administration of IL-2 at high dose in patients with metastatic RCC or advanced melanoma achieved significant clinical responses, the treatment was subsequently approved by the FDA [93]. However, the toxic profile of this regimen, including frequent grade 3 and 4 adverse effects, remains a hindrance for IL-2-based monotherapy [94]. IL-2 is also widely used for ex vivo expansion of tumor-infiltrating lymphocytes (TILs) or CAR-T cells in adaptive T cell therapy [95]. Of importance, with TILs usually being exhausted or dysfunctional, low-dose IL-2 can augment the antitumor response of CD8+ T cells [1]. A phase II clinical trial has shown promising results of using low-dose IL-2 following adoptive TILs transfer in patients with metastatic melanoma [96]. Still, there are several limitations that preclude this therapeutic success, including IL-2 induced AICD and preferential expansion of Treg cells.

Meanwhile, IL-15 has been recognized as a promising antitumor immunotherapeutic agent for its superior role in the stimulation of NK cells and T cells, persistence of CD8+ memory T cells and inhibition of AICD. Administration of exogenous IL-15 suppressed tumor growth in various preclinical models [97]. The first in-human clinical trial of recombinant human IL-15 (rhIL-15) was performed with intravenous bolus infusion to patients with metastatic RCC and melanoma. A dramatic expansion of NK cells and memory CD8+ T cells was observed in patients’ peripheral blood, but also accompanied with dose-limiting toxicities [98]. Later research showed that continuous IV infusion of rhIL-15 had a robust effect on CD8+ T and NK cells, particularly the CD56 bright subset of NK cells. Signs of antitumor effects were noticed in several patients with advanced cancer, but the best response observed was stable disease [99]. In another small-scale outpatient clinical trial, IL-15 was subcutaneously (SC) injected into patients with advanced solid tumors. This treatment was well-tolerated and significantly increased the amount of circulating NK and CD8+ T cells. There was no objective clinical response observed in this trial, but several patients showed disease stabilization during the treatment [100]. Another clinical trial, combining IV or SC rhIL-15 with haploidentical NK therapy for refractory acute myeloid leukemia (AML), was conducted recently, with 30–40% of patients achieving clinical response [101]. These clinical trial results indicate that improved pharmacological profiles or combination strategies are required for clinical application of IL-15.

### 4.2. Translational Advances of Engineered Cytokines

Severe adverse events and preferential expansion of immunosuppressive Treg cells limit the therapeutic use of recombinant IL-2 in cancer; these drawbacks are potentially associated with signals through the high-affinity receptor complex. Considerable effort has been made to prevent the off-target effects on Treg cells via eliminating the interaction with IL-2Rα. For instance, a superkine for IL-2, also called “super 2”, was designed with increased binding affinity to IL-2Rβγ, which allows it to function irrespective of IL-2Rα expression. Compared to wild-type IL-2, the superkine elicited enhanced expression of cytotoxic T cells but less of Treg cells [102]. Some newly developed fusion proteins that comprise IL-2 and IL-2Rα, such as ALKS 4230, could selectively active the effector cells bearing intermediate receptors, and exhibit superior tumor control efficacy and less toxicity in mouse tumor models [103]. Pharmacokinetic profile modification of IL-2 has been attained by chimerization with antibodies that direct the cytokine to the TME, or though covalent binding with moieties such as PEG molecules. Cergutuzumab amunaleukin (CEA-IL2V, RG7813) was developed with an IL-2 variant fused to a specific antibody which enables it to target carcinoembryonic antigens (CEAs) in vivo [104], and it is being studied in a clinical trial in combination with atezolizumab (NCT02350673). RO7284755, an IL-2 variant immunocytokine, fused with an anti-PD1 moiety, is currently being evaluated in a phase I/II study for the safety and antitumor activity (NCT04303858).

NKTR-214 (also known as Bempegaldesleukin) is the second generation of an engineered IL-2 compound that has been mostly investigated and reached several clinical trials. The six releasable PEG chains of this modified cytokine could mask the region of IL-2 that interacts with IL-2Rα, mediating biasing activation of cytolytic effector cells [105]. In the first in-human phase I clinical study (NCT02869295), NKTR-214 was well-tolerated and showed remarked clinical activity in patients with advanced solid tumors [106]. Based on its encouraging curative efficacy, NKTR-214 is being investigated in various clinical trials in combination with immune checkpoint blockades (Table 1).

The efficacy of IL-15 monotherapy might be restrained by the limited expression of IL-15Rα in vivo, which is required for the trans-presentation of IL-15 to mediate biological activity. Due to the small molecular size, recombinant IL-15 has a relatively short half-life after administration. To address these issues, efforts are now turning into developing fusion proteins that are stably constructed, comprising IL-15 and IL-15Rα. Several studies revealed that these alternative therapeutic forms of IL-15 display better distribution and persistence, with superior therapeutic potential compare to native IL-15. In the case of recombinant protein RLI, the soluble sushi domain of IL-15Rα and IL-15 are bound via a flexible linker; this fusion protein displays high affinity towards the IL-15Rβγ, and it operates as a superagonist by enhancing bioavailability and efficiency in metastatic melanoma and colorectal cancer [107,108]. ALT-803, another pharmacological grade complex that encompasses the IL-15 superagonist variant (IL-15N72D) and dimeric IL-15Rα Su/Fc, has been shown to exert superior antitumor activity in mouse models [109] and has been evaluated in clinical trials for various malignancies (Table 2), as monotherapy or in combination with other approved antitumor agents. In its first-in-human phase I trial (NCT01885897), ALT-803 treatment was well-tolerated and clinical response was observed in 19% of evaluated patients with hematologic malignancy [110]. Another dose-escalation phase I trial was recently performed assessing the safety and efficacy of ALT-803 in combination with nivolumab in patients with advanced non-small cell lung cancer (NSCLC) (NCT02523469); no dose-limiting toxicities were recorded and 6 of 21 patients achieved an objective response. A continuous phase II recruitment of patients is ongoing [111]. 

To further augment the therapeutic index of IL-15, several immunocytokines have been developed with additional antibodies that target tumor-associated antigens to specifically redirect IL-15 to TME, or with conjugated antitumor monoclonal antibodies to improve ADCC (antibody-dependent cellular cytotoxicity). Based on the IL-15 superagonist RLI, the fusion of anti-CD20 (rituximab, RTX) or anti-GD2 antibody domains has strongly boosted its antitumor potency in the mouse model [112,113]. Preclinical studies on Sushi-IL15-Apo, a triple recombinant protein conjugated with polipoprotein A-I to bind to SR-BI on tumor cells, displayed improved ADCC activity against a tumor of this chimeric protein [114]. A modified molecular 2B8T2M, with ALT-803 fused to rituximab, exhibited stronger antitumor activity through improved tumor recognition, efficient effector cell stimulation and enhanced ADCC [115]. Recently, a group of researchers designed a mimetic of IL-2 using a novel computational strategy, known as neoleukin-2/15 (Neo-2/15). This re-engineered protein is capable of binding IL2-Rβγ with high affinity but lacks the binding site for IL-2Rα or IL-15Rα, leading to the preferential expansion of cytotoxic CD8+T cells. When used in the mouse cancer model, either as monotherapy or as in combination with adoptive cell therapy, Neo-2/15 exhibited a potent capacity of tumor control, with unnoticeable immunogenicity and minor toxicity [116]. These studies showcase the translational potential for the next-generation cytokine therapeutics of IL-2 and IL-15.

### 4.3. Combinatorial Immune Strategies with IL-2 and IL-15

#### 4.3.1. Immune Checkpoint Inhibitors

Even with the advances of modified formulation of IL-2 and IL-15, the overall clinical response rate of monotherapy is still relatively low, thus more efforts are concentrated on exploring synergistic combinations with other therapeutic agents. Immune checkpoint inhibitors (ICIs), particularly the PD-1/PD-L1 blockade, have revolutionized the cancer immunotherapy in the past decade. The remarkable clinical efficacies of ICIs lead to the approval from the FDA for the treatment of various malignancies, such as NSCLC, melanoma and RCC, which provide the rationale for potential combinations with modified cytokines. Several studies have indicated that combined IL-2 therapy with anti-PD-1/PD-L1 antibodies significantly enhanced CD8+ T cell responses in the mouse model [37,117,118]. When used in combination, the IL-15 superagonist, RLI, has been shown to induce a stronger antitumor effect of PD-1 antagonists [119]. In line with this, an in vitro study in breast cancer suggested that the combination with IL-2 or IL-15 could boost the lytic activity against tumor cells, therefore augmenting the therapeutic efficacy of Avelumab [120]. Furthermore, the result of a phase I/II trial (PIVOT-02), a dose-escalation study evaluating the tolerability and immune activity of NKTR-214 plus Nivolumab in patients with advanced solid tumors (NCT02983045), indicated that the combination was well-tolerated and exhibited encouraging clinical efficacy regardless of baseline PD-L1 status [121]. Currently, several therapeutic strategies combining ICIs with IL-2 or IL-15 are being evaluated in clinical trials for safety and efficacy, and some of these trials have shown promising preliminary results (Table 1 and Table 2).

#### 4.3.2. Adoptive Cell Therapy

Cellular immunotherapy, also known as adoptive cell therapy (ACT), is another strategy to modify the immune system and provide new options to patients with cancer. ACT takes advantage of the natural ability of immune cells and could be deployed in different ways, including ACT with tumor-infiltrating lymphocytes (TILs), with T cells engineered with modified T cell receptor (TCR) or with chimeric antigen receptor (CAR) modified T cells, and with NK cells. The impressive efficacy results of anti-CD19 CAR-T cell therapy for hematological malignancies have led to its commercial approval by the FDA [122]. IL-2 has been extensively used for the in vitro expansion and in vivo persistence of adoptively transferred CAR-T cells or TILs for ACT [123]. However, due to its unique role to activate NK cells and CD8+ T cells without inducing Treg cells, IL-15 may potentially be superior to IL-2. The combinations of cytokine and ACT are currently being evaluated in multiple clinical trials in a variety of cancer types, including with different doses of IL-2 (NCT02414945, NCT04052334) or using IL-15 and IL-2 as an adjuvant for NK cell infusion (NCT03669172, NCT03213964). ACT also could be optimized by integration of the cytokine gene into the lentiviral vector that encodes the CARs, such as the anti-CD19 CAR-T or CAR-NK cells incorporated with IL-15 and inducible caspase-9 suicide genes (iC9/CAR.19/15) [124,125], anti-GD2 CAR-T cells with an IL-15 gene to target neuroblastoma (GD2.CAR/15) [126], and IL-15 trispecific killer engager (TriKE) containing single-chain Fv against CD16 and CD33 (as known as 161533) [127,128]. These constantly evolving engineered immune cells and combinations may provide an avenue for improving adoptive cell therapies in solid tumors.

#### 4.3.3. Other Combination Therapies

Apart from the combination options mentioned above, other approaches have been designed using clinical approved antitumor agents to synergize with IL-2 or IL-15, including agents to augment ADCC such as rituximab (NCT 02384954) or alemtuzumab (NCT 02689453), in combination with intravesical Bacillus Calmette-Guerin (BCG) for the treatment of bladder cancer (NCT02138734, NCT03022825), or combined with stereotactic body radiotherapy (SBRT) (NCT02306954, NCT01884961). It has been demonstrated that the combination of IL-15 and AKT inhibitor promoted the expansion of hepatocellular cancer TILs with elevated cytotoxic potential [129]. Additionally, IL-15 is also being widely explored in combination with antitumor vaccines. In a mouse model with established AML, vaccination with 32Dp210-IL-15/IL-15Rα/CD80 demonstrated 80% survival [130]. The measles virus Schwarz strain (MeVac) vector encoding with IL-2 has shown more immune activation, and MeVac with FmIL-15 promoted NK and T cell infiltration in mouse tumor models [131]. Together, these synergistic combination strategies will help to notably optimize the therapeutic use of IL-2 and IL-15.

## 5. Conclusions and Perspective

In summary, as members of the common γ chain receptor family of cytokines, IL-2 and IL-15 are potent but complicated immune modulators. Given their ability to expand and activate effector T cells and NK cells, cytokine therapy based on IL-2 or IL-15 provides alternative immunotherapy choices to patients following the traditional cancer treatments. Meanwhile, due to the redundant cytokine signaling and pleiotropic responses, these cytokines also display double-edged functions such as inducing immunosuppressive Treg cells or mediating apoptosis, which underscores the importance of exploring the complexity of cytokines in order to enhance the therapeutic efficacy and dampen adverse side effects. To achieve these goals, efforts have been dedicated to modifying recombinant cytokines using contemporary biotechnologies to amplify their antitumor efficacy while minimizing the toxicity. Our emerging knowledge of the biology of IL-2 and IL-15 and their receptor system also enables the development of rational strategies for improved clinical application in malignant diseases. Modified cytokines such as superagonists, immunocytokines, or even mimic proteins built de novo by computer approaches are now being evaluated; some of these have already presented encouraging responses in both preclinical and clinical settings. Another massively investigated approach to optimize the therapeutic use of IL-2 and IL-15 is combinatory treatment, employing cytokine therapy in combination with other antitumor agents such as ICIs, tumor-targeting monoclonal antibodies and BCG, with chemotherapy or radiotherapy, or as an adjuvant in adoptive cell therapies. In particular, due to the unique role of IL-15 in the maintenance of NK and CD8+ memory T cells, as well as in promoting the infiltration and persistence of effector cells within the TME, it is possible that IL-15 might be of great value in the development of immunotherapy against solid tumors.

With multiple relevant clinical trials being conducted, the results of these potential synergistic activities are eagerly expected, which will undoubtedly provide more insight into the future clinical practice. However, there are still many obstacles that have to be overcome to fully realize the therapeutic potential of IL-2 and IL-15, and a lot of them still lie beyond the scope of this review. Currently, the two key directions for the improvement of cytokine therapy are modulation of cytokines and rational combination strategies, but it is difficult to predict which approach will lead to superior clinical benefits in cancer patients. Still, clinical trials are required to define the usage with additional works on the safety issues in the implementation of IL-2 and IL-15. It is hoped that the effectiveness of IL-2 and IL-15 immunotherapy will continue to evolve from future advances.

## Figures and Tables

**Table 1:** Examples of ongoing clinical trials with IL-2.

Drug	Interventions	Conditions	Status	Phases	Enrollment	NCT no.
rhIL-2	IL-2 + BCG	Cutaneous Metastatic Melanoma	Not yet recruiting	II/III	100	NCT03928275
	IL-2+ Pembrolizumab + Radiotherapy	Advanced Solid Tumors	Recruiting	I/II	45	NCT03474497
	HD IL-2 + Nivolumab	Metastatic Melanoma, RCC	Recruiting	II	25	NCT03991130
	HD IL-2 + Radiation (SBRT)	Metastatic Melanoma, RCC	Recruiting	II	/	NCT02306954, NCT01884961
Immunocytokine	RO7284755 + Atezolizumab	Advanced Solid Tumors	Recruiting	I	440	NCT04303858
	RO6874281 + Pembrolizumab	Metastatic Melanoma	Recruiting	I	150	NCT03875079
	RO6874281 + Atezolizumab	Advanced Solid Tumors	Recruiting	II	322	NCT03386721
	RO6874281 + Trastuzumab + Cetuximab	Solid Tumors	Recruiting	I	205	NCT02627274
	ALKS 4230 + Pembrolizumab	Advanced Solid Tumors	Recruiting	I/II	/	NCT02799095, NCT04144517, NCT03861793
NKTR-214	NKTR-214 + Pembrolizumab	Advanced Solid tumors	Recruiting	I/II	135	NCT03138889
	NKTR-214 + Nivolumab	Sarcoma, Melanoma, Urothelial Cancer, RCC, Bladder cancer	Active or recruiting	II or III	/	NCT03282344, NCT03635983, NCT03785925, NCT04410445, NCT03729245, NCT04209114,
	NKTR-214 + Nivolumab + Ipilimumab	Advanced Solid tumors	Active, not recruiting	I/II	557	NCT02983045
	NKTR-214 + Nivolumab + SBRT	Prostate Cancer	Recruiting	I	45	NCT03835533
	NKTR-214 + Avelumab ± Talazoparib/Enzalutamide	SCCHN, mCRPC	Recruiting	II	127	NCT04052204
	NKTR-214 + VB10.NEO	Advanced Solid Tumors	Recruiting	I/II	65	NCT03548467
	NKTR-214 + NKTR-262 + nivolumab	Advanced Solid Tumors	Recruiting	I/II	393	NCT03435640
Adoptive Cell Therapy	IL-2+ FATE-NK100	Ovarian Cancer	Recruiting	I	16	NCT03213964
	IL-2 + IOV-2001	CLL, SLL	Recruiting	I/II	70	NCT04155710
	Low-dose IL-2 + TILs	Pleural Mesothelioma	Recruiting	I/II	10	NCT02414945
	High-dose IL-2 + TILs	Sarcoma, Advanced Solid tumors	Active or Recruiting	I	15/24	NCT04052334, NCT03991741
	IL-2 + TILs + Pembrolizumab	Metastatic Melanoma	Recruiting	II	170	NCT02621021

rhIL-2: recombinant human IL-2; BCG: Bacillus Calmette-Guérin; RCC: renal cell carcinoma; SBRT: stereotactic body radiotherapy; SCCHN: squamous cell carcinoma of the head and neck; mCRPC: metastatic castration resistant prostate cancer; CLL: chronic lymphocytic leukemia; SLL: small lymphocytic leukemia. TILs: tumor-infiltrating lymphocytes.

**Table 2:** Examples of ongoing clinical trials with IL-15.

Drug	Interventions	Conditions	Status	Phases	Enrollment	NCT No.
rhIL-15	rhIL-15 + Avelumab	T-cell Malignancies, Renal Carcinoma	Recruiting	I/II	25	NCT03905135, NCT04150562
	rhIL-15 + Mogamulizumab	T-cell Malignancies	Recruiting	I	20	NCT04185220
	rhIL-15 + Obinutuzumab	Chronic Lymphocyte Leukemia	Recruiting	I	24	NCT03759184
	rhIL-15 + Ipilimumab + Nivolumab	Refractory Solid Tumors	Recruiting	I	50	NCT03388632
	s.c. rhIL-15 + Alemtuzumab	T-cell Malignancies	Recruiting	I	30	NCT02689453
RTX-240	RTX-240 monotherapy	Adult Solid Tumor	Recruiting	I/II	172	NCT04372706
BJ-001	BJ-001 + Pembrolizumab	Advanced/Metastatic Solid Tumors	Recruiting	I	92	NCT04294576
NIZ985	NIZ985 + PDR001	Metastatic Solid Tumors	Recruiting	I	110	NCT02452268
	NIZ985 + Spartalizumab	Solid Tumors and Lymphoma	Recruiting	I	68	NCT04261439
ALT-803	ALT-803 monotherapy	Hematologic Malignancy	Active, not recruiting	I/II	61	NCT01885897
	ALT-803 + BCG	Non-muscle Invasive Bladder Cancer	Recruiting	I/II	596/160	NCT02138734, NCT03022825
	ALT-803 + Rituximab	B Cell Non-Hodgkin Lymphoma	Active, not recruiting	I/II	86	NCT02384954
	ALT-803 + Nivolumab	Non-small Cell Lung Cancer	Active, not recruiting	I/II	58	NCT02523469
	ALT-803 + Pembrolizumab/Nivolumab/Atezolizumab	Advanced Cancer	Recruiting	II	611	NCT03228667
	ALT-803 + Elotuzumab + Expanded Natural Killer (ENK) Cells	Multiple Myeloma	Recruiting	II	10	NCT03003728
N-803	N-803 + haNK™ + Avelumab	Merkel Cell Carcinoma	Recruiting	II	43	NCT03853317
	N-803 + CIML NK cell infusion + Ipilimumab	Head and Neck Squamous Cell Carcinoma	Recruiting	I	12	NCT04290546
Adoptive Cell Therapy/Engineered Cell	Donor IL-15-stimulated NK cells infusion	Acute Leukemia	Recruiting	I/II	3	NCT03669172
	IL-2 or IL-7/IL-15 pretreated CD19 cells	B Cell Lymphoma	Not yet recruiting	IV	10	NCT02992834
	iC9.GD2.CAR.IL-15 T-cells	Neuroblastoma	Recruiting	I	18	NCT03721068
	CD19-CD8-CD28-CD3zeta-CAR-mbIL15-HER1t T Cells	Lymphoblastic Leukemia	Not yet recruiting	I	12	NCT03579888
	GINAKIT Cells (GD2.CAR.IL-15 NKT Cells)	Neuroblastoma	Recruiting	I	24	NCT03294954
	GTB-3550 (CD16/IL-15/CD33) Tri-Specific Killer Engager (TriKE™)	High Risk Heme Malignancies	Recruiting	I/II	60	NCT03214666
	iC9/CAR.19/IL15-transduced CB-NK cells + AP1903	Lymphoid Malignancies	Recruiting	I/II	36	NCT03056339

rhIL-15: recombinant human IL-15; BCG: Bacillus Calmette-Guérin; CIML: Cytokine-induced memory-like; s.c. subcutaneous.

## References

[1] Leonard W.J., Lin J.X., O’Shea J.J. (2019). The γ(c) Family of Cytokines: Basic Biology to Therapeutic Ramifications. Immunity.

[2] Waldmann T.A. (2006). The biology of interleukin-2 and interleukin-15: Implications for cancer therapy and vaccine design. Nat. Rev. Immunol..

[3] Read K.A., Powell M.D., McDonald P.W., Oestreich K.J. (2016). IL-2, IL-7, and IL-15: Multistage regulators of CD4(+) T helper cell differentiation. Exp. Hematol..

[4] Marks-Konczalik J., Dubois S., Losi J.M., Sabzevari H., Yamada N., Feigenbaum L., Waldmann T.A., Tagaya Y. (2000). IL-2-induced activation-induced cell death is inhibited in IL-15 transgenic mice. Proc. Natl. Acad. Sci. USA.

[5] Mimura K., Kua L.F., Shimasaki N., Shiraishi K., Nakajima S., Siang L.K., Shabbir A., So J., Yong W.P., Kono K. (2017). Upregulation of thioredoxin-1 in activated human NK cells confers increased tolerance to oxidative stress. Cancer Immunol. Immunother. CII.

[6] Yang Y., Neo S.Y., Chen Z., Cui W., Chen Y., Guo M., Wang Y., Xu H., Kurzay A., Alici E. (2020). Thioredoxin activity confers resistance against oxidative stress in tumor-infiltrating NK cells. J. Clin. Investig..

[7] Kaur N., Naga O.S., Norell H., Al-Khami A.A., Scheffel M.J., Chakraborty N.G., Voelkel-Johnson C., Mukherji B., Mehrotra S. (2011). T cells expanded in presence of IL-15 exhibit increased antioxidant capacity and innate effector molecules. Cytokine.

[8] Rosenberg S.A. (2014). IL-2: The First Effective Immunotherapy for Human Cancer. J. Immunol..

[9] Morgan D.A., Ruscetti F.W., Gallo R. (1976). Selective in vitro growth of T lymphocytes from normal human bone marrows. Science.

[10] Liao W., Lin J.X., Leonard W.J. (2013). Interleukin-2 at the crossroads of effector responses, tolerance, and immunotherapy. Immunity.

[11] Paliard X., de Waal Malefijt R., Yssel H., Blanchard D., Chrétien I., Abrams J., de Vries J., Spits H. (1988). Simultaneous production of IL-2, IL-4, and IFN-gamma by activated human CD4+ and CD8+ T cell clones. J. Immunol..

[12] Granucci F., Vizzardelli C., Pavelka N., Feau S., Persico M., Virzi E., Rescigno M., Moro G., Ricciardi-Castagnoli P. (2001). Inducible IL-2 production by dendritic cells revealed by global gene expression analysis. Nat. Immunol..

[13] Hershko A.Y., Suzuki R., Charles N., Alvarez-Errico D., Sargent J.L., Laurence A., Rivera J. (2011). Mast cell interleukin-2 production contributes to suppression of chronic allergic dermatitis. Immunity.

[14] Yui M.A., Sharp L.L., Havran W.L., Rothenberg E.V. (2004). Preferential activation of an IL-2 regulatory sequence transgene in TCR gamma delta and NKT cells: Subset-specific differences in IL-2 regulation. J. Immunol..

[15] Crellin N.K., Trifari S., Kaplan C.D., Satoh-Takayama N., Di Santo J.P., Spits H. (2010). Regulation of cytokine secretion in human CD127(+) LTi-like innate lymphoid cells by Toll-like receptor 2. Immunity.

[16] Owen D.L., Mahmud S.A., Vang K.B., Kelly R.M., Blazar B.R., Smith K.A., Farrar M.A. (2018). Identification of Cellular Sources of IL-2 Needed for Regulatory T Cell Development and Homeostasis. J. Immunol..

[17] Gringhuis S.I., de Leij L.F., Verschuren E.W., Borger P., Vellenga E. (1997). Interleukin-7 upregulates the interleukin-2-gene expression in activated human T lymphocytes at the transcriptional level by enhancing the DNA binding activities of both nuclear factor of activated T cells and activator protein-1. Blood.

[18] Müller M.R., Rao A. (2010). NFAT, immunity and cancer: A transcription factor comes of age. Nat. Rev. Immunol..

[19] Kim H.P., Imbert J., Leonard W.J. (2006). Both integrated and differential regulation of components of the IL-2/IL-2 receptor system. Cytokine Growth Factor Rev..

[20] Mondino A., Whaley C.D., DeSilva D.R., Li W., Jenkins M.K., Mueller D.L. (1996). Defective transcription of the IL-2 gene is associated with impaired expression of c-Fos, FosB, and JunB in anergic T helper 1 cells. J. Immunol..

[21] Lindstein T., June C.H., Ledbetter J.A., Stella G., Thompson C.B. (1989). Regulation of lymphokine messenger RNA stability by a surface-mediated T cell activation pathway. Science.

[22] Grabstein K.H., Eisenman J., Shanebeck K., Rauch C., Srinivasan S., Fung V., Beers C., Richardson J., Schoenborn M.A., Ahdieh M. (1994). Cloning of a T cell growth factor that interacts with the beta chain of the interleukin-2 receptor. Science.

[23] Bamford R.N., Grant A.J., Burton J.D., Peters C., Kurys G., Goldman C.K., Brennan J., Roessler E., Waldmann T.A. (1994). The interleukin (IL) 2 receptor beta chain is shared by IL-2 and a cytokine, provisionally designated IL-T, that stimulates T-cell proliferation and the induction of lymphokine-activated killer cells. Proc. Natl. Acad. Sci. USA.

[24] Waldmann T.A., Tagaya Y. (1999). The multifaceted regulation of interleukin-15 expression and the role of this cytokine in NK cell differentiation and host response to intracellular pathogens. Annu. Rev. Immunol..

[25] Armitage R.J., Macduff B.M., Eisenman J., Paxton R., Grabstein K.H. (1995). IL-15 has stimulatory activity for the induction of B cell proliferation and differentiation. J. Immunol..

[26] Waldmann T.A., Miljkovic M.D., Conlon K.C. (2020). Interleukin-15 (dys)regulation of lymphoid homeostasis: Implications for therapy of autoimmunity and cancer. J. Exp. Med..

[27] Fehniger T.A., Caligiuri M.A. (2001). Interleukin 15: Biology and relevance to human disease. Blood.

[28] Tagaya Y., Kurys G., Thies T.A., Losi J.M., Azimi N., Hanover J.A., Bamford R.N., Waldmann T.A. (1997). Generation of secretable and nonsecretable interleukin 15 isoforms through alternate usage of signal peptides. Proc. Natl. Acad. Sci. USA.

[29] Tan X., Lefrançois L. (2006). Novel IL-15 isoforms generated by alternative splicing are expressed in the intestinal epithelium. Genes Immun..

[30] Gaggero A., Azzarone B., Andrei C., Mishal Z., Meazza R., Zappia E., Rubartelli A., Ferrini S. (1999). Differential intracellular trafficking, secretion and endosomal localization of two IL-15 isoforms. Eur. J. Immunol..

[31] Waldmann T.A. (2015). The Shared and Contrasting Roles of IL2 and IL15 in the Life and Death of Normal and Neoplastic Lymphocytes: Implications for Cancer Therapy. Cancer Immunol. Res..

[32] Cao X., Kozak C.A., Liu Y.J., Noguchi M., O’Connell E., Leonard W.J. (1993). Characterization of cDNAs encoding the murine interleukin 2 receptor (IL-2R) gamma chain: Chromosomal mapping and tissue specificity of IL-2R gamma chain expression. Proc. Natl. Acad. Sci. USA.

[33] Gruss H.J., Scott C., Rollins B.J., Brach M.A., Herrmann F. (1996). Human fibroblasts express functional IL-2 receptors formed by the IL-2R alpha- and beta-chain subunits: Association of IL-2 binding with secretion of the monocyte chemoattractant protein-1. J. Immunol..

[34] Lin J.X., Leonard W.J. (1997). The immediate-early gene product Egr-1 regulates the human interleukin-2 receptor beta-chain promoter through noncanonical Egr and Sp1 binding sites. Mol. Cell. Biol..

[35] Ramirez K., Chandler K.J., Spaulding C., Zandi S., Sigvardsson M., Graves B.J., Kee B.L. (2012). Gene deregulation and chronic activation in natural killer cells deficient in the transcription factor ETS1. Immunity.

[36] Sowell R.T., Goldufsky J.W., Rogozinska M., Quiles Z., Cao Y., Castillo E.F., Finnegan A., Marzo A.L. (2017). IL-15 Complexes Induce Migration of Resting Memory CD8 T Cells into Mucosal Tissues. J. Immunol..

[37] Hong C., Luckey M.A., Ligons D.L., Waickman A.T., Park J.Y., Kim G.Y., Keller H.R., Etzensperger R., Tai X., Lazarevic V. (2014). Activated T cells secrete an alternatively spliced form of common γ-chain that inhibits cytokine signaling and exacerbates inflammation. Immunity.

[38] Gesbert F., Malardé V., Dautry-Varsat A. (2005). Ubiquitination of the common cytokine receptor gammac and regulation of expression by an ubiquitination/deubiquitination machinery. Biochem. Biophys. Res. Commun..

[39] Driesen J., Popov A., Schultze J.L. (2008). CD25 as an immune regulatory molecule expressed on myeloid dendritic cells. Immunobiology.

[40] Nakamura Y., Russell S.M., Mess S.A., Friedmann M., Erdos M., Francois C., Jacques Y., Adelstein S., Leonard W.J. (1994). Heterodimerization of the IL-2 receptor beta- and gamma-chain cytoplasmic domains is required for signalling. Nature.

[41] Takeshita T., Asao H., Ohtani K., Ishii N., Kumaki S., Tanaka N., Munakata H., Nakamura M., Sugamura K. (1992). Cloning of the gamma chain of the human IL-2 receptor. Science.

[42] Rubin L.A., Nelson D.L. (1990). The soluble interleukin-2 receptor: Biology, function, and clinical application. Ann. Intern. Med..

[43] Murakami S. (2004). Soluble interleukin-2 receptor in cancer. Front. Biosci..

[44] Karim A.F., Eurelings L.E.M., Bansie R.D., van Hagen P.M., van Laar J.A.M., Dik W.A. (2018). Soluble Interleukin-2 Receptor: A Potential Marker for Monitoring Disease Activity in IgG4-Related Disease. Mediat. Inflamm..

[45] Waldmann T.A. (2002). The IL-2/IL-15 receptor systems: Targets for immunotherapy. J. Clin. Immunol..

[46] Ring A.M., Lin J.-X., Feng D., Mitra S., Rickert M., Bowman G.R., Pande V.S., Li P., Moraga I., Spolski R. (2012). Mechanistic and structural insight into the functional dichotomy between IL-2 and IL-15. Nat. Immunol..

[47] Lodolce J.P., Burkett P.R., Boone D.L., Chien M., Ma A. (2001). T cell-independent interleukin 15Ralpha signals are required for bystander proliferation. J. Exp. Med..

[48] Dubois S., Mariner J., Waldmann T.A., Tagaya Y. (2002). IL-15Ralpha recycles and presents IL-15 In trans to neighboring cells. Immunity.

[49] Wuest S.C., Edwan J.H., Martin J.F., Han S., Perry J.S., Cartagena C.M., Matsuura E., Maric D., Waldmann T.A., Bielekova B. (2011). A role for interleukin-2 trans-presentation in dendritic cell-mediated T cell activation in humans, as revealed by daclizumab therapy. Nat. Med..

[50] Friedmann M.C., Migone T.S., Russell S.M., Leonard W.J. (1996). Different interleukin 2 receptor beta-chain tyrosines couple to at least two signaling pathways and synergistically mediate interleukin 2-induced proliferation. Proc. Natl. Acad. Sci. USA.

[51] Lin J.X., Li P., Liu D., Jin H.T., He J., Ata Ur Rasheed M., Rochman Y., Wang L., Cui K., Liu C. (2012). Critical Role of STAT5 transcription factor tetramerization for cytokine responses and normal immune function. Immunity.

[52] Lin J.X., Du N., Li P., Kazemian M., Gebregiorgis T., Spolski R., Leonard W.J. (2017). Critical functions for STAT5 tetramers in the maturation and survival of natural killer cells. Nat. Commun..

[53] Ross S.H., Cantrell D.A. (2018). Signaling and Function of Interleukin-2 in T Lymphocytes. Annu. Rev. Immunol..

[54] Miyazaki T., Liu Z.J., Kawahara A., Minami Y., Yamada K., Tsujimoto Y., Barsoumian E.L., Permutter R.M., Taniguchi T. (1995). Three distinct IL-2 signaling pathways mediated by bcl-2, c-myc, and lck cooperate in hematopoietic cell proliferation. Cell.

[55] Ross S.H., Rollings C., Anderson K.E., Hawkins P.T., Stephens L.R., Cantrell D.A. (2016). Phosphoproteomic Analyses of Interleukin 2 Signaling Reveal Integrated JAK Kinase-Dependent and -Independent Networks in CD8(+) T Cells. Immunity.

[56] Pereno R., Giron-Michel J., Gaggero A., Cazes E., Meazza R., Monetti M., Monaco E., Mishal Z., Jasmin C., Indiveri F. (2000). IL-15/IL-15Ralpha intracellular trafficking in human melanoma cells and signal transduction through the IL-15Ralpha. Oncogene.

[57] Keating N., Nicholson S.E. (2019). SOCS-mediated immunomodulation of natural killer cells. Cytokine.

[58] Delconte R.B., Kolesnik T.B., Dagley L.F., Rautela J., Shi W., Putz E.M., Stannard K., Zhang J.G., Teh C., Firth M. (2016). CIS is a potent checkpoint in NK cell-mediated tumor immunity. Nat. Immunol..

[59] Roediger B., Kyle R., Tay S.S., Mitchell A.J., Bolton H.A., Guy T.V., Tan S.Y., Forbes-Blom E., Tong P.L., Köller Y. (2015). IL-2 is a critical regulator of group 2 innate lymphoid cell function during pulmonary inflammation. J. Allergy Clin. Immunol..

[60] Robinette M.L., Bando J.K., Song W., Ulland T.K., Gilfillan S., Colonna M. (2017). IL-15 sustains IL-7R-independent ILC2 and ILC3 development. Nat. Commun..

[61] Klose C.S., Blatz K., d’Hargues Y., Hernandez P.P., Kofoed-Nielsen M., Ripka J.F., Ebert K., Arnold S.J., Diefenbach A., Palmer E. (2014). The transcription factor T-bet is induced by IL-15 and thymic agonist selection and controls CD8αα(+) intraepithelial lymphocyte development. Immunity.

[62] Lenardo M.J. (1996). Fas and the art of lymphocyte maintenance. J. Exp. Med..

[63] Zhang X., Sun S., Hwang I., Tough D.F., Sprent J. (1998). Potent and selective stimulation of memory-phenotype CD8+ T cells in vivo by IL-15. Immunity.

[64] Schluns K.S., Klonowski K.D., Lefrançois L. (2004). Transregulation of memory CD8 T-cell proliferation by IL-15Ralpha+ bone marrow-derived cells. Blood.

[65] Vang K.B., Yang J., Mahmud S.A., Burchill M.A., Vegoe A.L., Farrar M.A. (2008). IL-2, -7, and -15, but not thymic stromal lymphopoeitin, redundantly govern CD4+Foxp3+ regulatory T cell development. J. Immunol..

[66] Burchill M.A., Yang J., Vogtenhuber C., Blazar B.R., Farrar M.A. (2007). IL-2 receptor beta-dependent STAT5 activation is required for the development of Foxp3+ regulatory T cells. J. Immunol..

[67] Fontenot J.D., Rasmussen J.P., Gavin M.A., Rudensky A.Y. (2005). A function for interleukin 2 in Foxp3-expressing regulatory T cells. Nat. Immunol..

[68] Li M.O., Rudensky A.Y. (2016). T cell receptor signalling in the control of regulatory T cell differentiation and function. Nat. Rev. Immunol..

[69] Boyman O., Sprent J. (2012). The role of interleukin-2 during homeostasis and activation of the immune system. Nat. Rev. Immunol..

[70] Hirakawa M., Matos T.R., Liu H., Koreth J., Kim H.T., Paul N.E., Murase K., Whangbo J., Alho A.C., Nikiforow S. (2016). Low-dose IL-2 selectively activates subsets of CD4(+) Tregs and NK cells. JCI Insight.

[71] Zhao H., Nguyen H., Kang J. (2005). Interleukin 15 controls the generation of the restricted T cell receptor repertoire of gamma delta intestinal intraepithelial lymphocytes. Nat. Immunol..

[72] Van Acker H.H., Campillo-Davo D., Roex G., Versteven M., Smits E.L., Van Tendeloo V.F. (2018). The role of the common gamma-chain family cytokines in γδ T cell-based anti-cancer immunotherapy. Cytokine Growth Factor Rev..

[73] Leclercq G., Debacker V., de Smedt M., Plum J. (1996). Differential effects of interleukin-15 and interleukin-2 on differentiation of bipotential T/natural killer progenitor cells. J. Exp. Med..

[74] Arneja A., Johnson H., Gabrovsek L., Lauffenburger D.A., White F.M. (2014). Qualitatively different T cell phenotypic responses to IL-2 versus IL-15 are unified by identical dependences on receptor signal strength and duration. J. Immunol..

[75] Leonard W.J. (2001). Cytokines and immunodeficiency diseases. Nat. Rev. Immunol..

[76] Sadlack B., Löhler J., Schorle H., Klebb G., Haber H., Sickel E., Noelle R.J., Horak I. (1995). Generalized autoimmune disease in interleukin-2-deficient mice is triggered by an uncontrolled activation and proliferation of CD4+ T cells. Eur. J. Immunol..

[77] Sadlack B., Merz H., Schorle H., Schimpl A., Feller A.C., Horak I. (1993). Ulcerative colitis-like disease in mice with a disrupted interleukin-2 gene. Cell.

[78] Schorle H., Holtschke T., Hünig T., Schimpl A., Horak I. (1991). Development and function of T cells in mice rendered interleukin-2 deficient by gene targeting. Nature.

[79] Willerford D.M., Chen J., Ferry J.A., Davidson L., Ma A., Alt F.W. (1995). Interleukin-2 receptor α chain regulates the size and content of the peripheral lymphoid compartment. Immunity.

[80] Lodolce J.P., Boone D.L., Chai S., Swain R.E., Dassopoulos T., Trettin S., Ma A. (1998). IL-15 receptor maintains lymphoid homeostasis by supporting lymphocyte homing and proliferation. Immunity.

[81] Kennedy M.K., Glaccum M., Brown S.N., Butz E.A., Viney J.L., Embers M., Matsuki N., Charrier K., Sedger L., Willis C.R. (2000). Reversible defects in natural killer and memory CD8 T cell lineages in interleukin 15-deficient mice. J. Exp. Med..

[82] Cornish G.H., Sinclair L.V., Cantrell D.A. (2006). Differential regulation of T-cell growth by IL-2 and IL-15. Blood.

[83] Klebanoff C.A., Finkelstein S.E., Surman D.R., Lichtman M.K., Gattinoni L., Theoret M.R., Grewal N., Spiess P.J., Antony P.A., Palmer D.C. (2004). IL-15 enhances the in vivo antitumor activity of tumor-reactive CD8+ T cells. Proc. Natl. Acad. Sci. USA.

[84] Beltra J.C., Bourbonnais S., Bédard N., Charpentier T., Boulangé M., Michaud E., Boufaied I., Bruneau J., Shoukry N.H., Lamarre A. (2016). IL2Rβ-dependent signals drive terminal exhaustion and suppress memory development during chronic viral infection. Proc. Natl. Acad. Sci. USA.

[85] Heon E.K., Wulan H., Macdonald L.P., Malek A.O., Braunstein G.H., Eaves C.G., Schattner M.D., Allen P.M., Alexander M.O., Hawkins C.A. (2015). IL-15 induces strong but short-lived tumor-infiltrating CD8 T cell responses through the regulation of Tim-3 in breast cancer. Biochem. Biophys. Res. Commun..

[86] Wagner J.A., Rosario M., Romee R., Berrien-Elliott M.M., Schneider S.E., Leong J.W., Sullivan R.P., Jewell B.A., Becker-Hapak M., Schappe T. (2017). CD56bright NK cells exhibit potent antitumor responses following IL-15 priming. J. Clin. Investig..

[87] Marçais A., Cherfils-Vicini J., Viant C., Degouve S., Viel S., Fenis A., Rabilloud J., Mayol K., Tavares A., Bienvenu J. (2014). The metabolic checkpoint kinase mTOR is essential for IL-15 signaling during the development and activation of NK cells. Nat. Immunol..

[88] Mao Y., van Hoef V., Zhang X., Wennerberg E., Lorent J., Witt K., Masvidal L., Liang S., Murray S., Larsson O. (2016). IL-15 activates mTOR and primes stress-activated gene expression leading to prolonged antitumor capacity of NK cells. Blood.

[89] Felices M., Lenvik A.J., McElmurry R., Chu S., Hinderlie P., Bendzick L., Geller M.A., Tolar J., Blazar B.R., Miller J.S. (2018). Continuous treatment with IL-15 exhausts human NK cells via a metabolic defect. JCI Insight.

[90] Ndhlovu L.C., Lopez-Vergès S., Barbour J.D., Jones R.B., Jha A.R., Long B.R., Schoeffler E.C., Fujita T., Nixon D.F., Lanier L.L. (2012). Tim-3 marks human natural killer cell maturation and suppresses cell-mediated cytotoxicity. Blood.

[91] Viel S., Marcais A., Guimaraes F.S., Loftus R., Rabilloud J., Grau M., Degouve S., Djebali S., Sanlaville A., Charrier E. (2016). TGF-beta inhibits the activation and functions of NK cells by repressing the mTOR pathway. Sci. Signal..

[92] Dubois S., Shou W., Haneline L.S., Fleischer S., Waldmann T.A., Müller J.R. (2003). Distinct pathways involving the FK506-binding proteins 12 and 12.6 underlie IL-2-versus IL-15-mediated proliferation of T cells. Proc. Natl. Acad. Sci. USA.

[93] Rosenberg S.A., Yang J.C., Topalian S.L., Schwartzentruber D.J., Weber J.S., Parkinson D.R., Seipp C.A., Einhorn J.H., White D.E. (1994). Treatment of 283 consecutive patients with metastatic melanoma or renal cell cancer using high-dose bolus interleukin 2. JAMA.

[94] Berraondo P., Sanmamed M.F., Ochoa M.C., Etxeberria I., Aznar M.A., Luis Perez-Gracia J., Rodriguez-Ruiz M.E., Ponz-Sarvise M., Castanon E., Melero I. (2019). Cytokines in clinical cancer immunotherapy. Br. J. Cancer.

[95] Dudley M.E., Wunderlich J.R., Robbins P.F., Yang J.C., Hwu P., Schwartzentruber D.J., Topalian S.L., Sherry R., Restifo N.P., Hubicki A.M. (2002). Cancer regression and autoimmunity in patients after clonal repopulation with antitumor lymphocytes. Science.

[96] Nguyen L.T., Saibil S.D., Sotov V., Le M.X., Khoja L., Ghazarian D., Bonilla L., Majeed H., Hogg D., Joshua A.M. (2019). Phase II clinical trial of adoptive cell therapy for patients with metastatic melanoma with autologous tumor-infiltrating lymphocytes and low-dose interleukin-2. Cancer Immunol. Immunother..

[97] Waldmann T.A. (2018). Cytokines in Cancer Immunotherapy. Cold Spring Harb. Perspect. Biol..

[98] Conlon K.C., Lugli E., Welles H.C., Rosenberg S.A., Fojo A.T., Morris J.C., Fleisher T.A., Dubois S.P., Perera L.P., Stewart D.M. (2015). Redistribution, Hyperproliferation, Activation of Natural Killer Cells and CD8 T Cells, and Cytokine Production During First-in-Human Clinical Trial of Recombinant Human Interleukin-15 in Patients With Cancer. J. Clin. Oncol..

[99] Conlon K.C., Potter E.L., Pittaluga S., Lee C.-C.R., Miljkovic M.D., Fleisher T.A., Dubois S., Bryant B.R., Petrus M., Perera L.P. (2019). IL15 by Continuous Intravenous Infusion to Adult Patients with Solid Tumors in a Phase I Trial Induced Dramatic NK-Cell Subset Expansion. Clin. Cancer Res..

[100] Miller J.S., Morishima C., McNeel D.G., Patel M.R., Kohrt H.E.K., Thompson J.A., Sondel P.M., Wakelee H.A., Disis M.L., Kaiser J.C. (2018). A First-in-Human Phase I Study of Subcutaneous Outpatient Recombinant Human IL15 (rhIL15) in Adults with Advanced Solid Tumors. Clin. Cancer Res..

[101] Cooley S., He F., Bachanova V., Vercellotti G.M., DeFor T.E., Curtsinger J.M., Robertson P., Grzywacz B., Conlon K.C., Waldmann T.A. (2019). First-in-human trial of rhIL-15 and haploidentical natural killer cell therapy for advanced acute myeloid leukemia. Blood Adv..

[102] Levin A.M., Bates D.L., Ring A.M., Krieg C., Lin J.T., Su L., Moraga I., Raeber M.E., Bowman G.R., Novick P. (2012). Exploiting a natural conformational switch to engineer an interleukin-2 ‘superkine’. Nature.

[103] Lopes J.E., Fisher J.L., Flick H.L., Wang C., Sun L., Ernstoff M.S., Alvarez J.C., Losey H.C. (2020). ALKS 4230: A novel engineered IL-2 fusion protein with an improved cellular selectivity profile for cancer immunotherapy. J. Immunother. Cancer.

[104] Klein C., Waldhauer I., Nicolini V.G., Freimoser-Grundschober A., Nayak T., Vugts D.J., Dunn C., Bolijn M., Benz J., Stihle M. (2017). Cergutuzumab amunaleukin (CEA-IL2v), a CEA-targeted IL-2 variant-based immunocytokine for combination cancer immunotherapy: Overcoming limitations of aldesleukin and conventional IL-2-based immunocytokines. Oncoimmunology.

[105] Charych D.H., Hoch U., Langowski J.L., Lee S.R., Addepalli M.K., Kirk P.B., Sheng D., Liu X., Sims P.W., VanderVeen L.A. (2016). NKTR-214, an Engineered Cytokine with Biased IL2 Receptor Binding, Increased Tumor Exposure, and Marked Efficacy in Mouse Tumor Models. Clin. Cancer Res..

[106] Bentebibel S.E., Hurwitz M.E., Bernatchez C., Haymaker C., Hudgens C.W., Kluger H.M., Tetzlaff M.T., Tagliaferri M.A., Zalevsky J., Hoch U. (2019). A First-in-Human Study and Biomarker Analysis of NKTR-214, a Novel IL2Rβγ-Biased Cytokine, in Patients with Advanced or Metastatic Solid Tumors. Cancer Discov..

[107] Mortier E., Quéméner A., Vusio P., Lorenzen I., Boublik Y., Grötzinger J., Plet A., Jacques Y. (2006). Soluble interleukin-15 receptor alpha (IL-15R alpha)-sushi as a selective and potent agonist of IL-15 action through IL-15R beta/gamma. Hyperagonist IL-15 x IL-15R alpha fusion proteins. J. Biol. Chem..

[108] Bessard A., Sole V., Bouchaud G., Quemener A., Jacques Y. (2009). High antitumor activity of RLI, an interleukin-15 (IL-15)-IL-15 receptor alpha fusion protein, in metastatic melanoma and colorectal cancer. Mol. Cancer Ther..

[109] Rhode P.R., Egan J.O., Xu W., Hong H., Webb G.M., Chen X., Liu B., Zhu X., Wen J., You L. (2016). Comparison of the Superagonist Complex, ALT-803, to IL15 as Cancer Immunotherapeutics in Animal Models. Cancer Immunol. Res..

[110] Romee R., Cooley S., Berrien-Elliott M.M., Westervelt P., Verneris M.R., Wagner J.E., Weisdorf D.J., Blazar B.R., Ustun C., DeFor T.E. (2018). First-in-human phase 1 clinical study of the IL-15 superagonist complex ALT-803 to treat relapse after transplantation. Blood.

[111] Wrangle J.M., Velcheti V., Patel M.R., Garrett-Mayer E., Hill E.G., Ravenel J.G., Miller J.S., Farhad M., Anderton K., Lindsey K. (2018). ALT-803, an IL-15 superagonist, in combination with nivolumab in patients with metastatic non-small cell lung cancer: A non-randomised, open-label, phase 1b trial. Lancet Oncol..

[112] Vincent M., Bessard A., Cochonneau D., Teppaz G., Solé V., Maillasson M., Birklé S., Garrigue-Antar L., Quéméner A., Jacques Y. (2013). Tumor targeting of the IL-15 superagonist RLI by an anti-GD2 antibody strongly enhances its antitumor potency. Int. J. Cancer.

[113] Vincent M., Teppaz G., Lajoie L., Solé V., Bessard A., Maillasson M., Loisel S., Béchard D., Clémenceau B., Thibault G. (2014). Highly potent anti-CD20-RLI immunocytokine targeting established human B lymphoma in SCID mouse. MAbs.

[114] Ochoa M.C., Minute L., López A., Pérez-Ruiz E., Gomar C., Vasquez M., Inoges S., Etxeberria I., Rodriguez I., Garasa S. (2018). Enhancement of antibody-dependent cellular cytotoxicity of cetuximab by a chimeric protein encompassing interleukin-15. Oncoimmunology.

[115] Liu B., Kong L., Han K., Hong H., Marcus W.D., Chen X., Jeng E.K., Alter S., Zhu X., Rubinstein M.P. (2016). A Novel Fusion of ALT-803 (Interleukin (IL)-15 Superagonist) with an Antibody Demonstrates Antigen-specific Antitumor Responses. J. Biol. Chem..

[116] Silva D.A., Yu S., Ulge U.Y., Spangler J.B., Jude K.M., Labão-Almeida C., Ali L.R., Quijano-Rubio A., Ruterbusch M., Leung I. (2019). De novo design of potent and selective mimics of IL-2 and IL-15. Nature.

[117] West E.E., Jin H.T., Rasheed A.U., Penaloza-Macmaster P., Ha S.J., Tan W.G., Youngblood B., Freeman G.J., Smith K.A., Ahmed R. (2013). PD-L1 blockade synergizes with IL-2 therapy in reinvigorating exhausted T cells. J. Clin. Investig..

[118] Rahimi Kalateh Shah Mohammad G., Ghahremanloo A., Soltani A., Fathi E., Hashemy S.I. (2020). Cytokines as potential combination agents with PD-1/PD-L1 blockade for cancer treatment. J. Cell. Physiol..

[119] Desbois M., Le Vu P., Coutzac C., Marcheteau E., Beal C., Terme M., Gey A., Morisseau S., Teppaz G., Boselli L. (2016). IL-15 Trans-Signaling with the Superagonist RLI Promotes Effector/Memory CD8(+) T Cell Responses and Enhances Antitumor Activity of PD-1 Antagonists. J. Immunol..

[120] Juliá E.P., Amante A., Pampena M.B., Mordoh J., Levy E.M. (2018). Avelumab, an IgG1 anti-PD-L1 Immune Checkpoint Inhibitor, Triggers NK Cell-Mediated Cytotoxicity and Cytokine Production Against Triple Negative Breast Cancer Cells. Front. Immunol..

[121] Diab A., Tannir N.M., Bentebibel S.E., Hwu P., Papadimitrakopoulou V., Haymaker C., Kluger H.M., Gettinger S.N., Sznol M., Tykodi S.S. (2020). Bempegaldesleukin (NKTR-214) plus Nivolumab in Patients with Advanced Solid Tumors: Phase I Dose-Escalation Study of Safety, Efficacy, and Immune Activation (PIVOT-02). Cancer Discov..

[122] June C.H., O’Connor R.S., Kawalekar O.U., Ghassemi S., Milone M.C. (2018). CAR T cell immunotherapy for human cancer. Science.

[123] Guedan S., Ruella M., June C.H. (2019). Emerging Cellular Therapies for Cancer. Annu. Rev. Immunol..

[124] Hoyos V., Savoldo B., Quintarelli C., Mahendravada A., Zhang M., Vera J., Heslop H.E., Rooney C.M., Brenner M.K., Dotti G. (2010). Engineering CD19-specific T lymphocytes with interleukin-15 and a suicide gene to enhance their anti-lymphoma/leukemia effects and safety. Leukemia.

[125] Liu E., Tong Y., Dotti G., Shaim H., Savoldo B., Mukherjee M., Orange J., Wan X., Lu X., Reynolds A. (2018). Cord blood NK cells engineered to express IL-15 and a CD19-targeted CAR show long-term persistence and potent antitumor activity. Leukemia.

[126] Chen Y., Sun C., Landoni E., Metelitsa L., Dotti G., Savoldo B. (2019). Eradication of Neuroblastoma by T Cells Redirected with an Optimized GD2-Specific Chimeric Antigen Receptor and Interleukin-15. Clin. Cancer Res..

[127] Sarhan D., Brandt L., Felices M., Guldevall K., Lenvik T., Hinderlie P., Curtsinger J., Warlick E., Spellman S.R., Blazar B.R. (2018). 161533 TriKE stimulates NK-cell function to overcome myeloid-derived suppressor cells in MDS. Blood Adv..

[128] Vallera D.A., Felices M., McElmurry R., McCullar V., Zhou X., Schmohl J.U., Zhang B., Lenvik A.J., Panoskaltsis-Mortari A., Verneris M.R. (2016). IL15 Trispecific Killer Engagers (TriKE) Make Natural Killer Cells Specific to CD33+ Targets While Also Inducing Persistence, In Vivo Expansion, and Enhanced Function. Clin. Cancer Res..

[129] Xu B., Yuan L., Chen G., Li T., Zhou J., Zhang C., Qin P., Muthana M.M., Wang S., Du X. (2019). S-15 in combination of Akt inhibitor promotes the expansion of CD45RA(-)CCR7(+) tumor infiltrating lymphocytes with high cytotoxic potential and downregulating PD-1(+)Tim-3(+) cells as well as regulatory T cells. Cancer Cell Int..

[130] Shi Y., Dincheva-Vogel L., Ayemoba C.E., Fung J.P., Bergamaschi C., Pavlakis G.N., Farzaneh F., Gaensler K.M.L. (2018). IL-15/IL-15Rα/CD80-expressing AML cell vaccines eradicate minimal residual disease in leukemic mice. Blood Adv..

[131] Backhaus P.S., Veinalde R., Hartmann L., Dunder J.E., Jeworowski L.M., Albert J., Hoyler B., Poth T., Jaeger D., Ungerechts G. (2019). Immunological Effects and Viral Gene Expression Determine the Efficacy of Oncolytic Measles Vaccines Encoding IL-12 or IL-15 Agonists. Viruses.

